# The effect of low dose amphetamine in rotenone-induced toxicity in a mice model of Parkinson’s disease

**DOI:** 10.22038/ijbms.2020.45175.10524

**Published:** 2020-09

**Authors:** Omar M.E. Abdel-Salam, Safaa M. Youssef Morsy, Eman R. Youness, Noha N. Yassen, Amany A Sleem

**Affiliations:** 1 Department of Toxicology and Narcotics, National Research Centre, Cairo, Egypt; 2 Department of Medical Biochemistry, National Research Centre, Cairo, Egypt; 3 Department of Pathology, National Research Centre, Cairo, Egypt; 4 Department of Pharmacology, National Research Centre, Cairo, Egypt

**Keywords:** Amphetamine, Anti-oxidant capacity, Neuroprotection, Parkinson’s disease, Reactive oxygen species, Rotenone

## Abstract

**Objective(s):**

The effects of low dose amphetamine on oxidative stress and rotenone-induced neurotoxicity and liver injury were examined *in vivo* in a mice model of Parkinson’s disease.

**Materials and Methods:**

Male mice were treated with rotenone (1.5 mg/kg, every other day for two weeks, subcutaneously). Mice received either the vehicle or amphetamine intraperitoneally at doses of 0.5, 1.0, or 2.0 mg/kg. Oxidative stress was assessed by measurement of the lipid peroxidation product malondialdehyde (MDA), nitric oxide (NO), total anti-oxidant capacity (TAC), and paraoxonase-1 (PON-1) activity in the brain and liver. In addition, brain concentrations of nuclear factor kappa B (NF-κB) and tyrosine hydroxylase were determined and histopathology and Bax/Bcl-2 immunohistochemistry were performed.

**Results:**

The levels of lipid peroxidation and NO were increased and TAC and PON-1 were decreased significantly compared with vehicle-injected control mice. There were also significantly increased NF-κB and decreased tyrosine hydroxylase in the brain following rotenone administration. These changes were significantly attenuated by amphetamine. Rotenone caused neurodegenerative changes in the substantia nigra, cerebral cortex, and hippocampus. The liver showed degenerative changes in hepatocytes and infiltration of Kupffer cells. Bax/Bcl2 ratio was significantly increased in brain and liver tissues. Amphetamine prevented these histopathological changes and the increase in apoptosis evoked by rotenone.

**Conclusion:**

These results suggest that low dose amphetamine exerts anti-oxidant and anti-apoptotic effects, protects against rotenone-induced neurodegeneration, and could prevent neuronal cell degeneration in Parkinson’s disease.

## Introduction

Parkinson’s disease (PD) is a common neurodegenerative disorder affecting 1% of the elderly population over the age of 65. The incidence increases with advancing age (1, 2) and is expected to rise with consequent increase in social and economic burden (3). PD is caused by the progressive loss of the dopamine neurons in the substantia nigra pars compacta (SNpc) of the midbrain basal ganglia (4). The function of the basal ganglia is to control motor behavior. These nuclei are also important for cognition (5). The loss of pigmented neurons in the SNpc results in a marked deficit of dopamine content in the striatum, which is related to the duration and the clinical severity of the disease (6). The typical clinical features are bradykinesia (or akinesia), resting tremor, rigidity, and postural instability (7). 

The common underlying mechanisms that may mediate the damage to SNpc dopamine neurons are increased production of reactive oxygen species and neuroinflammation (8, 9). An imbalance between the rate in which reactive oxygen species are produced and the capacity of cellular anti-oxidants results in oxidative stress with consequent oxidative damage to the cell membrane lipids, proteins, enzymes, and DNA (10). Post-mortem studies reported that oxidative stress was substantially increased in the brain tissue of patients with PD compared with controls (11). There were also elevated levels of pro-inflammatory cytokines such as tumor necrosis factor-alpha (TNF-α) and interleukin 1beta (IL-1β) in the striatum and substantia nigra of these patients (12). 

Pesticides have been implicated in causing neurodegeneration in PD (13). Rotenone, a pesticide of plant origin, has been shown to cause PD-like features e.g., dopamine depletion, death of cells in the substantia nigra and striatum, Lewy bodies like deposits, and hypokinesia when injected into rats or mice (14,15). Rotenone causes neuronal cell death through an increase in oxidative stress. Rotenone increases the generation of reactive oxygen metabolites intracellularly (16). Being an inhibitor of mitochondrial complex I or NADH-ubiquinone reductase, the pesticide impairs mitochondrial function and this action is thought to result in increased generation of superoxide causing damage to mitochondria and initiating apoptosis (17, 18).

Presently, the treatment of PD is largely based on the dopamine precursor L-dopa (L-3, 4-dihydroxy-phenylalanine) administered together with carbidopa which acts to inhibit the peripheral decarboxylation of L-dopa, allow it to reach the brain in therapeutic amounts and provide symptomatic relief (19). Other drugs include dopamine receptor agonists, catechol O-methyl-transferase inhibitors, monoamine oxidase inhibitors, and amantadine (20). After several years of treatment and due to the continued loss of dopaminergic neurons, these drugs became less efficacious together with the emergence of L-dopa-induced motor complications of dyskinesia and motor fluctuations (21). Moreover, none of the available drugs prevent the progressive death of dopaminergic cells (22), thereby necessitating the development or search for new treatments capable of preventing or decreasing cell death.

Substituted phenylethylamine derivatives, amphetamines, that are structurally similar to dopamine and endogenous trace amines, represent a well-known group of compounds that potently affect psychomotor functions (23-25). Amphetamine (1-methyl-2-phenethylamine) is a psycho-stimulant that is approved by the FDA for treating attention deficit hyperactivity and narcolepsy (23). Amphetamines interact with plasma membrane monoamine transporters, including dopamine (DA) transporter (DAT), norepinephrine (NE) transporter (NET), and serotonin transporter (SERT). This complex interaction results in the transporter-dependent efflux of monoamines into extracellular space from intraneuronal stores, resulting in increased concentrations of monoamine neurotransmitters in the synaptic cleft (26). In rodents, the use of high doses of amphetamine or methamphetamine has been associated with damage to substantia nigra dopaminergic neurons and terminals in the striatum (27, 28). There are reports, however, which indicate a better motor recovery in cerebral ischemia and traumatic brain injury in rats treated with low dose amphetamines (2-5 mg/kg) (29, 30). 

In this study, using the rotenone-induced PD mice model, we investigated whether low doses of amphetamine could reduce the rotenone-induced degeneration of dopaminergic neurons. Rotenone causes fatty liver, vacuolar degeneration and hepatocyte apoptosis (31, 32). Therefore, we also studied the effect of amphetamines on liver injury evoked by the pesticide.

## Materials and Methods

***Animals***

Male Swiss albino mice (20-25 g: National Research Centre, Cairo) were used in the experiments. Mice were housed under a standard 12-hr light/dark cycle and had free access to food and water. 

***Drugs and Chemicals***

Rotenone and d-amphetamine sulfate were obtained from Sigma-Aldrich (St. Louis, Mo, USA). Rotenone was dissolved in 100% dimethyl sulfoxide. Amphetamine was diluted in physiological saline to obtain the necessary doses. Other chemicals and reagents were purchased from Sigma-Aldrich (St. Louis, Mo, USA) and were of analytical grade. 

***Study design***

Mice were randomly allocated into equal groups, six mice each. Mice received subcutaneous injections of rotenone 1.5 mg/kg every other day for two weeks combined with either saline (group 1) or amphetamines at doses of 0.5, 1.0, or 2.0 mg/kg (groups 2-4). Saline or amphetamines were intraperitoneally given at the time of rotenone injection. A 5^th^ group received only the vehicle (no rotenone) and served as a control. Thereafter, mice were euthanized by cervical decapitation under light ether anesthesia. The brain and liver of each mouse were then quickly removed, washed with ice-cold phosphate-buffered saline (PBS, pH 7.4), weighed, and stored at -80 ^°^C until the biochemical analyses were carried out. The tissues were homogenized in 0.1 M PBS at pH 7.4 to give a final concentration of 10 % w/v for the biochemical assays.

***Biochemical analyses***

*Determination of lipid peroxidation*

Malondialdehyde (MDA), an end product of lipid peroxidation was measured according to the method described previously (33). Thiobarbituric acid reactive substances (TBAS) react with thiobarbituric acid-forming TBA-MDA adduct and the absorbance is read at 532 nm using a spectrophotometer.

*Determination of nitric oxide*

Nitric oxide was measured using Griess reagent according to the literature (34). Nitrate is converted to nitrite by nitrate reductase. Griess reagent then converts nitrite to a deep purple azo compound. The absorbance is read at 540 nm using a spectrophotometer. Nitrite, a stable end-product of nitric oxide radical, is mostly used as an indicator for the production of nitric oxide. 

*Determination of total anti-oxidant capacity*

Total anti-oxidant capacity (TAC) in the supernatants was measured using a commercially available kit test (Biodiagnostics, Cairo, A.R.E.). This assay measures anti-oxidant capacity by the reaction of anti-oxidants in the sample with a defined amount of exogenously provided hydrogen peroxide. The anti-oxidants eliminate a certain amount of peroxide. The residual peroxide is determined colorimetrically by an enzymatic reaction that involves the conversion of 3, 5-dichloro-2-hydroxybenzenesulfate to a colored product (35).

*Determination of paraoxonase-1*

The arylesterase activity of PON-1 was determined by a colorimetric method using phenylacetate as a substrate. In this assay, PON-1 catalyzes the cleavage of phenylacetate resulting in phenol formation. The rate of formation of phenol was measured by monitoring the increase in absorbance at 270 nm and 25 ^°^C. The working mix consisted of 20 mM Tris/HCl buffer, pH 8.0, containing 1 mM CaCl_2_ and 4 mM phenylacetate as the substrate. Samples diluted 1:3 in buffer were added to the above mix and the changes in absorbance were recorded following a 20 sec lag time. One unit of arylesterase activity is equal to 1 μmole of phenol formed per min. The PON-1 activity is expressed in kU/L, based on the extinction coefficient of phenol of 1310 M^‒1^cm^‒1^. Blank samples containing water were used to correct for the spontaneous hydrolysis of phenylacetate (36).

***Quantification of nuclear factor kappa-B***

NOVA enzyme-linked immunosorbent assay (ELISA) kit from Bioneovan Co., Ltd. Beijing, China was used.

***Quantification of tyrosine hydroxylase***

Tyrosine hydroxylase was determined using NOVA human tyrosine hydroxylase (TH) ELISA kit (Bioneovan Co., Ltd. Beijing, China).

***Histopathological studies***

Livers and brains of all animals were dissected immediately after death. The specimens were then fixed in 10 % neutral-buffered formalin saline for 72 hr at least. Specimens were washed in tap water for half an hour and then dehydrated in ascending grades of alcohol, cleared in xylene, and embedded in paraffin. Serial sections of 5 μm thick were cut and stained with hematoxylin and eosin for histopathological investigation (37). Images were examined and photographed under a digital camera (Microscope Digital Camera DP70, Tokyo) and processed using Adobe Photoshop version 8.0.

***Immunohistochemistry for Bax and Bcl-2***

Formalin-fixed, paraffin-embedded tissue sections (5-µm thick) were de-paraffinized in xylene and rehydrated in graded alcohol. For antigen retrieval of Bx and Bcl-2, the slides were microwave boiled in citrate buffer (10 mmol/L, pH 6.0) for 7 min. Endogenous peroxidase activity was quenched with 3% hydrogen peroxide for 5 min. Non-specific binding of the primary antibodies was blocked by incubating the slides in 3% goat serum at room temperature for 1 hr in humidity chambers with the primary mouse monoclonal antibodies for Bax (Clone B9, Santa Cruz Biotechnology Inc, CA, USA) (1:200) and Bcl-2 (Clone 124, Roche Diagnostic Corporation, Indianapolis, IN, USA) (1:60). A biotin-streptavidin horseradish peroxidase detection kit was used as the secondary detection system (BioGenex). The biotinylated goat anti-mouse secondary and avidin-horseradish peroxidase label were each applied for 10 min. The antigen-antibody complex was recognized by incubating with the chromogen, diamino-benzidine Sigma, St Louis, MO) for 7 min. The slides were counterstained with hematoxylin for 1 min. Known positive controls were included in each staining run; negative controls were obtained by omitting the primary antibody. Slides were then dehydrated in graded alcohols, cleared in 3 xylene baths. 

***Quantitative analysis of Bax and Bcl-2***

Quantitative analysis of the percentage of cells that displayed the positive immunohistochemical stain for Bax and Bcl-2 was done with the use of a Leica Qwin 500 Image Analyzer (LEICA Imaging Systems Ltd, Cambridge, England). Bax/Bcl-2 ratio was calculated by dividing the area percentage of Bax (an apoptosis promoter) positive cells by the area percentage of Bcl-2 (an apoptosis inhibitor) positive cells. The percentage of positive cells gave the apoptotic body index (ABI) for each case (38).

***Statistical analysis***

Results are expressed as mean±SE. Data were statistically analyzed using one-way analysis of variance (ANOVA) followed by Duncan’s multiple range test for group comparison using SPSS software (SAS Institute Inc., Cary, NC). A probability value of less than 0.05 was considered statistically significant.

## Results

***Brain parameters***

*Oxidative stress*

**Figure 1:**
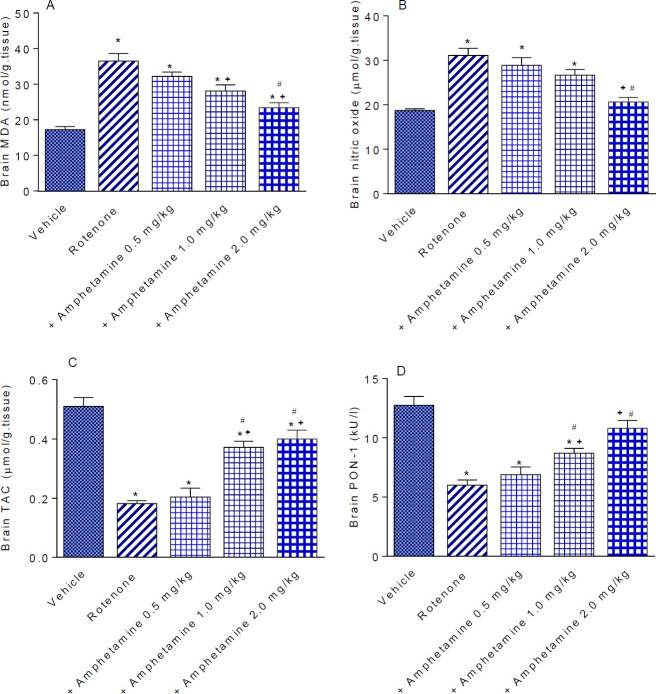
Effect of amphetamine treatment on brain malondialdehyde (MDA), nitric oxide, total anti-oxidant capacity (TAC), and paraoxonase-1 (PON-1) activity in rotenone-treated mice. *: *P<*0.05 vs vehicle. +: *P<*0.05 vs rotenone control. #: *P<*0.05 vs rotenone +amphetamine 0.5 mg/kg

Rotenone significantly increased brain MDA content by 112.2% compared with the vehicle-treated animals (36.5±2.1 vs 17.24±0.86 nmol/g tissue) (Figure 1A). Nitric oxide content increased by 66.3% (31.1±1.6 vs 18.7±0.86 µmol/g tissue) (Figure 1B) while a significant decrease in TAC by 64.3% was observed in rotenone-treated animals compared with their vehicle-treated counterparts (0.182±0.01 vs 0.51±0.03 µmol/g tissue) (Figure 1C).

Amphetamine given at doses of 1 or 2 mg/kg reduced MDA levels by 23.0% and 35.6% (28.1±1.7 and 23.4±1.4 vs 36.5±2.1 nmol/g tissue) (Figure 1A). Nitric oxide significantly decreased by 33.6% after amphetamine treatment at 2 mg/kg (20.65±1.0 vs 31.1±1.6 µmol/g tissue) (Figure 1B). Mice treated with amphetamine at 1 or 2 mg/kg showed significant increments in TAC by 104.4% and 120.0%, respectively (0.372±0.02 and 0.40±0.03 vs 0.182±0.01 µmol/g tissue) (Figure 1C).

*Paraoxonase-1*

The activity of PON-1 was decreased by 52.9% in rotenone treated mice compared with vehicle-treated controls (6.0±0.44 vs 12.74±0.75 kU/l). PON-1 activity increased by 45.2% and 80.0%, respectively following the administration of 1 or 2 mg/kg of amphetamine (8.71±0.41 and 10.8±0.67 vs 6.0±0.44 kU/l) (Figure 1D).

*Nuclear factor kappa-B*

**Figure 2:**
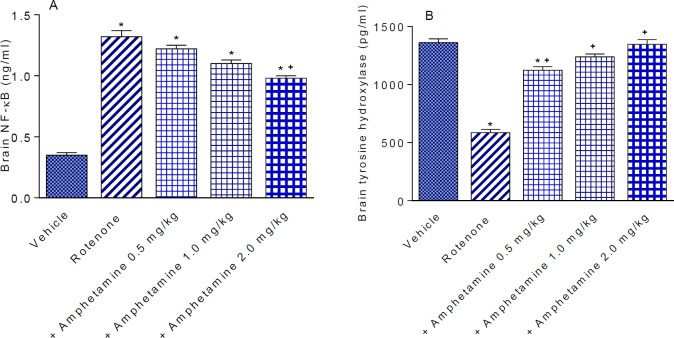
Effect of amphetamine treatment on the rotenone-induced changes in nuclear factor kappa-B (NF-κB) and tyrosine hydroxylase in mice brain. *: *P<*0.05 vs vehicle. +: *P<*0.05 vs rotenone control

The concentration of NF-κB in the vehicle treated group was 0.35±0.02 ng/ml. The concentration of NF-κB increased by 277.0% after rotenone administration (1.32±0.05 ng/ml). NF-κB significantly decreased by 25.8% by 2 mg/kg of amphetamine (0.98±0.02 vs 1.32±0.05 ng/ml)(Figure 2A).

*Tyrosine hydroxylase*

Rotenone produced a significant 57.0% decrease in striatal tyrosine hydroxylase content, compared with vehicle-treated control animals (585.1±28.0 vs 1362.2±30.7 pg/ml). Mice given amphetamine had a 92.0%, 111.5%, and 130.3% increase in tyrosine hydroxylase levels (1123.5±30.8, 1237.7±26.0 and 1347.3±39.9 vs 585.1±28.0 pg/ml). The effect of amphetamine was dose-dependent (Figure 2B). 

***Liver parameters***

*Oxidative stress*

**Figure 3:**
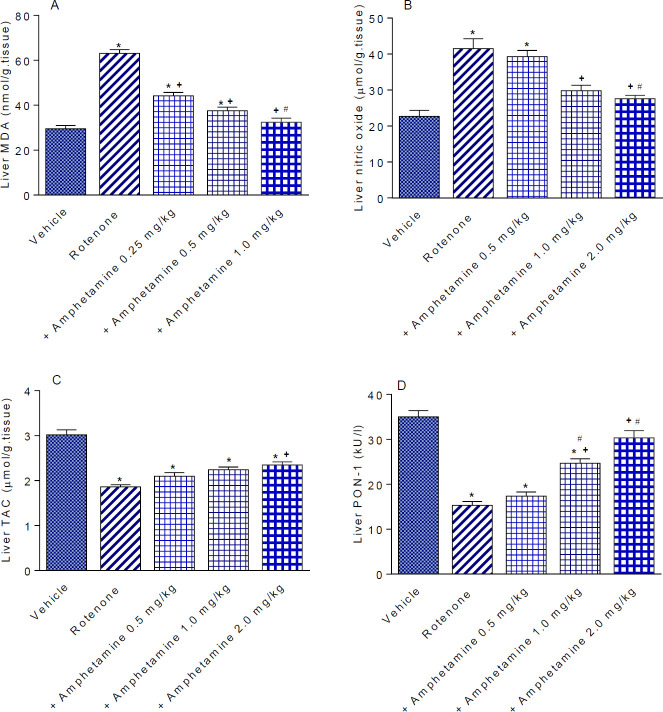
Effect of amphetamine treatment on liver malondialdehyde (MDA), nitric oxide, total antioxidant capacity (TAC), and paraoxonase-1 (PON-1) activity in rotenone-treated mice. *: P<0.05 vs vehicle. +: *P<*0.05 vs rotenone control. #: *P<*0.05 vs rotenone +amphetamine 0.5 mg/kg

Administration of rotenone resulted in a significantly increased MDA level by 113.8% compared with the vehicle control group (63.1±1.67 vs 29.51±1.45 nmol/g tissue) (Figure 3A). There was also significantly increased nitric oxide content by 80.7% (41.5±2.78 vs 22.96±1.66 µmol/g tissue) and decreased TAC by 38.4% (1.86±0.05 vs 3.02±0.11 µmol/g tissue) in the rotenone only group (Figures 3B&C).

The level of MDA decreased markedly after amphetamine administration by 30%, 40.5%, and 48%, respectively (44.16±1.51, 37.56±1.6, 32.8±1.84 vs 63.1±1.67 nmol/g tissue) (Figure 3A). Nitric oxide decreased by 28.2% and 33.5% by amphetamine at 1 or 2 mg/kg, respectively (29.8±1.53, 27.6±0.93 vs 41.5±2.78 µmol/g tissue) (Figure 3B). TAC was unchanged by amphetamine at doses of 0.5 or 1 mg/kg but increased by 26.3% in mice given 2 mg/kg amphetamine compared with the rotenone only group (2.35 0.07 vs 1.86 0.05 µmol/g tissue) (Figure 3C).

*Paraoxonase-1*

Paraoxonase-1 activity in the rotenone only group was significantly decreased by 56.4% compared with the vehicle-treated group (15.3±0.88 vs 35.1±1.4 kU/l). Administration of amphetamine at doses of 1 or 2 mg/kg significantly increased PON-1 activity by 61.4% and 98.7%, respectively, compared with the rotenone only-treated group (24.7±1.0 and 30.4±1.58 vs 15.3±0.88 kU/l) (Figure 3D).

***Histopathological results of the brain***

*Substantia nigra*

**Figure 4:**
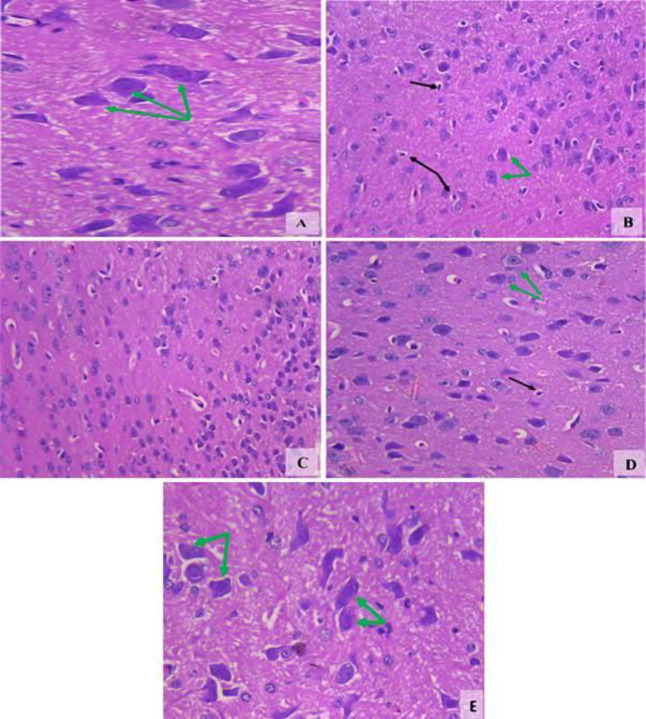
Representative photomicrographs of sections of the substantia nigra from (A) Vehicle. (B) Rotenone only. (C) Rotenone +amphetamine 0.5 mg/kg. (D) Rotenone+amphetamine 1 mg/kg. (E) Rotenone+amphetamine 2 mg/kg. Black arrows; degenerated neuronal cells. Green arrows; healthy dendritic pigmented neurons (H&E x 200 & 400)

The vehicle group showed a normal structure (Figure 4A). Rotenone resulted in decreased cellularity. Most neuronal cells showed degeneration and some neurons exhibited dense basophilic nuclei (Figure 4B). Mice treated with amphetamine at 0.5 mg/kg showed increased neuronal number but not well-formed cellular architecture (Figure 4C). Increased number of neurons and improved neuronal architecture were observed after treatment with 1 mg/kg of amphetamine (Figure 4D). Mice treated with 2 mg/kg amphetamine showed normal appearance of the substantia nigra (Figure 4E).

*Cerebral cortex*

**Figure 5:**
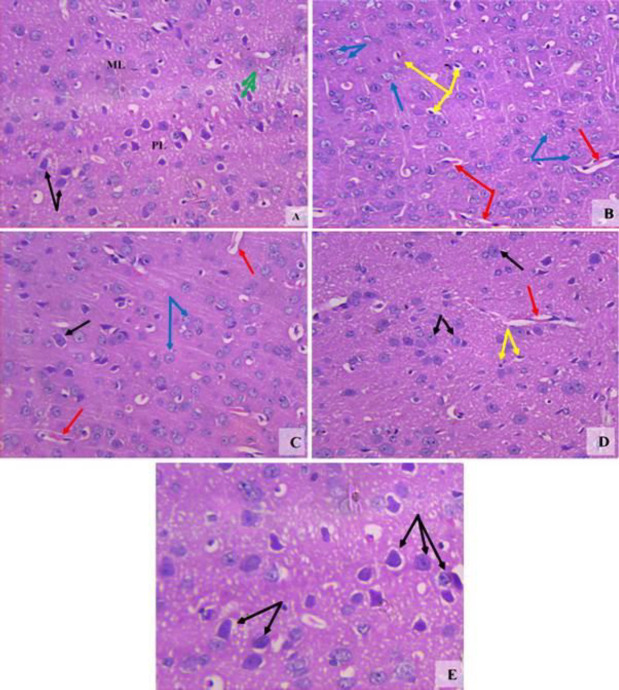
Representative photomicrographs of sections of the cerebral cortex from (A) Vehicle. (B) Rotenone only. (C) Rotenone+amphetamine 0.5 mg/kg. (D) Rotenone+amphetamine 1 mg/kg. (E) Rotenone+amphetamine 2 mg/kg. Abbreviations: ML; molecular layer, PL; pyramidal layer. Black arrows; normal neuronal cells. Green arrows; Purkinje cells. Yellow arrows; pyknotic deeply stained nuclei, Blue arrows; cells with nuclear karyolysis. Red arrows; congested cerebral vessels (H&E 200x & 400x)

The vehicle group showed the normal structure of the cerebral cortex (Figure 5A). Sections from the rotenone control group showed different forms of neuronal cells. Some neurons appeared with karyolytic nuclei and others with pyknotic deeply basophilic nuclei. There were also massively congested cerebral vessels (Figure 5B). Mice treated with amphetamine at 0.5 and 1 mg/kg showed dose-dependent improvement with minimal degenerated cells and congested vessels (Figures 5C&D). Mice treated with amphetamine at 2 mg/kg showed normal structure and architecture of the cerebral cortex with well-formed granular dendritic neuronal cells (Figure 5E).

*Hippocampus*

**Figure 6:**
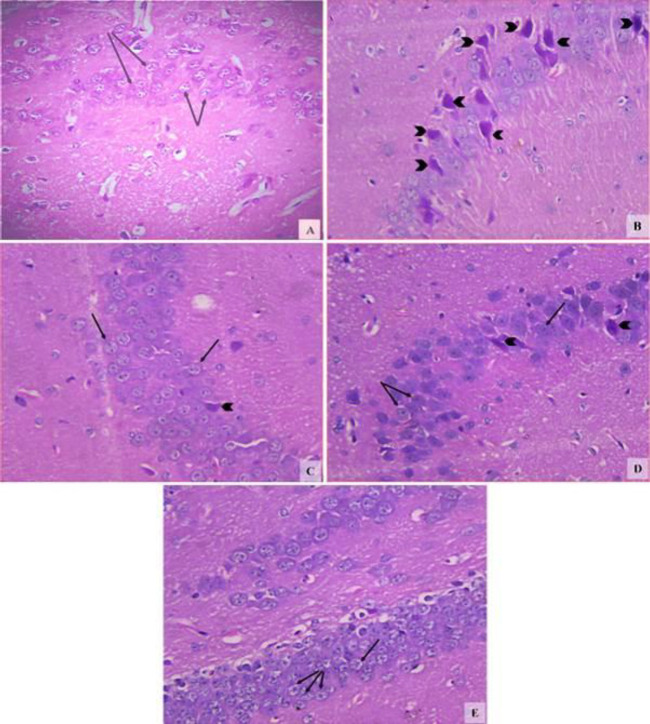
Representative photomicrographs of sections of hippocampus region from (A) Vehicle. (B) Rotenone only. (C) Rotenone+amphetamine 0.5 mg/kg. (D) Rotenone+amphetamine 1 mg/kg. (E) Rotenone+amphetamine 2 mg/kg. Black arrow heads; pyknotic nuclei. Black arrows; normally granular cells with rounded vesicular nuclei (H&E x400)

The hippocampal region of the vehicle group showed compact layers of small intact pyramidal cells with vesicular nuclei (Figure 6A). Rotenone resulted in neuronal cell loss with degenerative and necrotic changes as most of the cells had pyknotic nuclei (Figure 6B). A decrease in degenerative changes with minimal neuronal loss was observed after treatment with 0.5 and 1 mg/kg amphetamine as compared with the rotenone control group (Figures 6C&D). Mice treated with amphetamine at 2 mg/kg showed the normal structure of this region being formed of pyramidal cells with large vesicular nuclei (Figure 6E). 

***Brain Bax and Bcl2 immunoreactivities***

**Figure 7:**
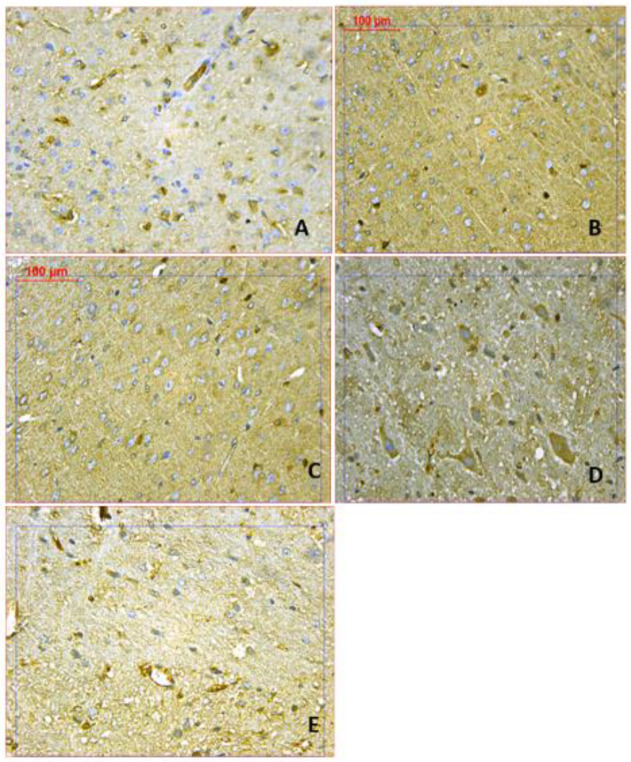
Representative photomicrographs of Bax immunoreactivities in the cortex from (A) Vehicle. (B) Rotenone only. (C) Rotenone+amphetamine 0.5 mg/kg. (D) Rotenone+amphetamine 1 mg/kg. (E) Rotenone+amphetamine 2 mg/kg (BAX 400x)

**Figure 8:**
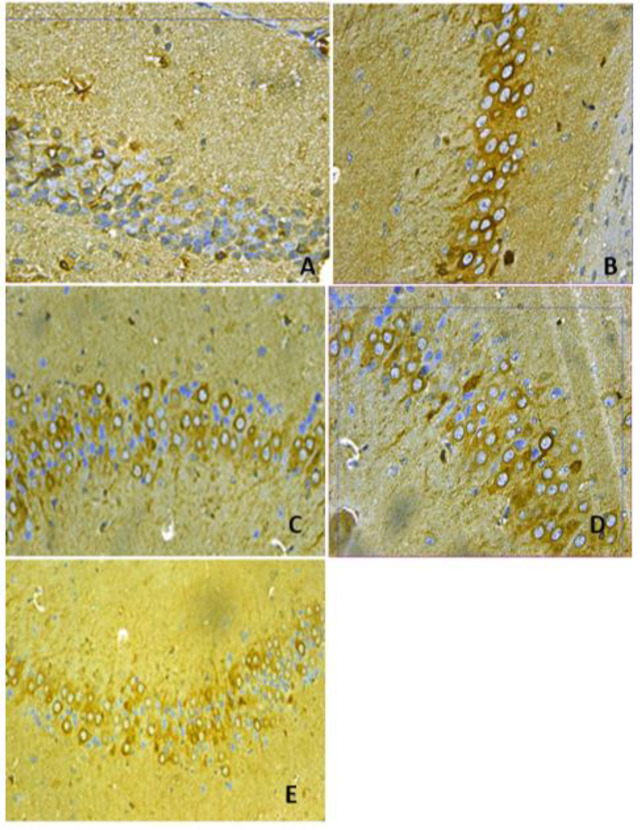
Representative photomicrographs of sections of hippocampus region stained by BAX from (A) Vehicle. (B) Rotenone only. (C) Rotenone+amphetamine 0.5 mg/kg. (D) Rotenone+amphetamine 1 mg/kg. (E) Rotenone+amphetamine 2 mg/kg (BAX 200x &400x)

**Figure 9:**
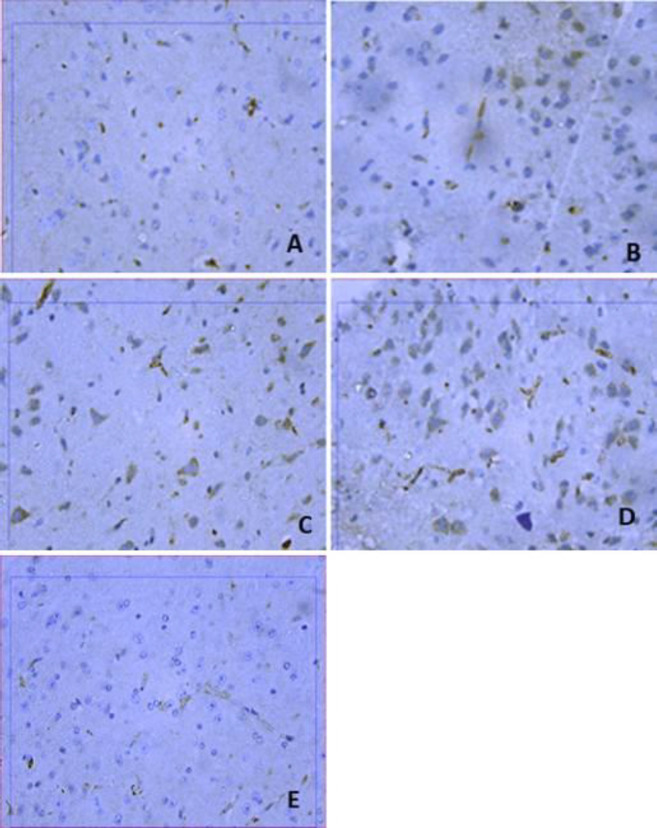
Representative photomicrographs of Bcl2 immunoreactivities in the cortex from (A) Vehicle. (B) Rotenone only. (C) Rotenone+amphetamine 0.5 mg/kg. (D) Rotenone+amphetamine 1 mg/kg. (E) Rotenone+amphetamine 2 mg/kg (Bcl 400x)

Rotenone caused a significant increase in immunostaining for Bax and a significant decrease in Bcl2 immunostaining compared with the vehicle group (Figures 7-9). Mice that received amphetamine showed a dose-dependent decrease in Bax-positive cells and an increase in Bcl-2-positive cells. There was a significant 1487.2% increase in the Bax/Bcl-2 ratio in the cerebral cortex in the rotenone only group compared with the vehicle group. Bax/Bcl-2 ratio decreased by 33.1%, 69.6%, and 87.1% in mice treated with amphetamine (Figures 7, 9, and Table 1). 

***Histopathological results of the liver***

**Figure 10:**
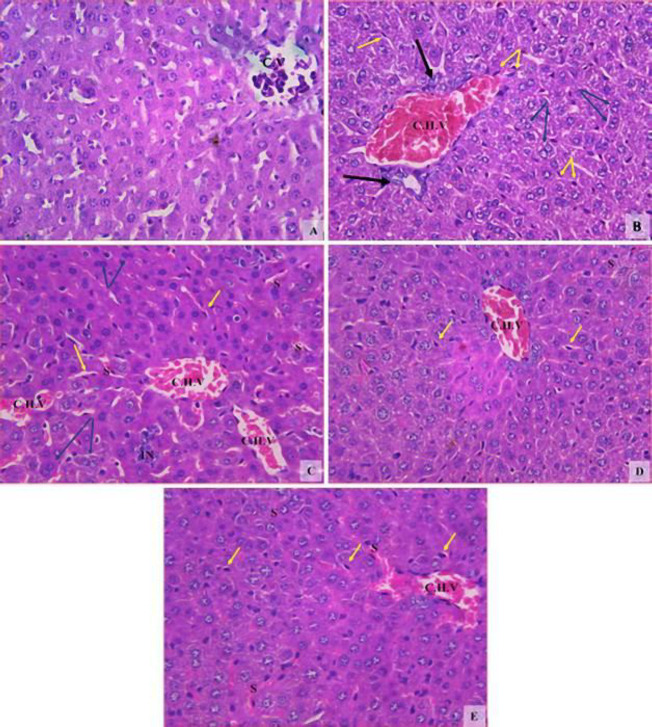
Representative photomicrographs of sections of liver tissue from (A) Vehicle. (B) Rotenone only. (C) Rotenone+amphetamine 0.5 mg/kg. (D) Rotenone+amphetamine 1 mg/kg. (E) Rotenone+amphetamine 2 mg/kg. Abbreviations: CV; central vein, CHV; congested hepatic vein, S; dilated congested sinusoids, IN; inflammatory cells. Yellow arrows; Kupffer cells. Blue arrows; binucleated hepatocytes. Black arrows; ductular hyperplasia (H&E x200)

The liver of the vehicle groups showed the normal characteristic architecture (Figure 10A). Rotenone induced hepatocyte degenerative changes with ruptured cell walls, most of them binuclear, with severe infiltration of Kupffer cells. There were also severely dilated congested hepatic veins with ductular hyperplasia (Figure 10B). Mice administered amphetamine at 0.5 mg/kg showed improved hepatocytes, decreased binuclear cells, and decreased Kupffer cells. However, multiple mildly dilated congested hepatic vessels dilated congested hepatic sinusoids, and focal inflammatory cells were present (Figure 10C). Mice given amphetamine at 1 or 2 mg/kg showed improvement in the histological picture with decreased dilatation and congestion of hepatic vessels and sinusoids (Figures 10D&E).

***Liver Bax and Bcl2 immunoreactivities***

**Figure 11.**
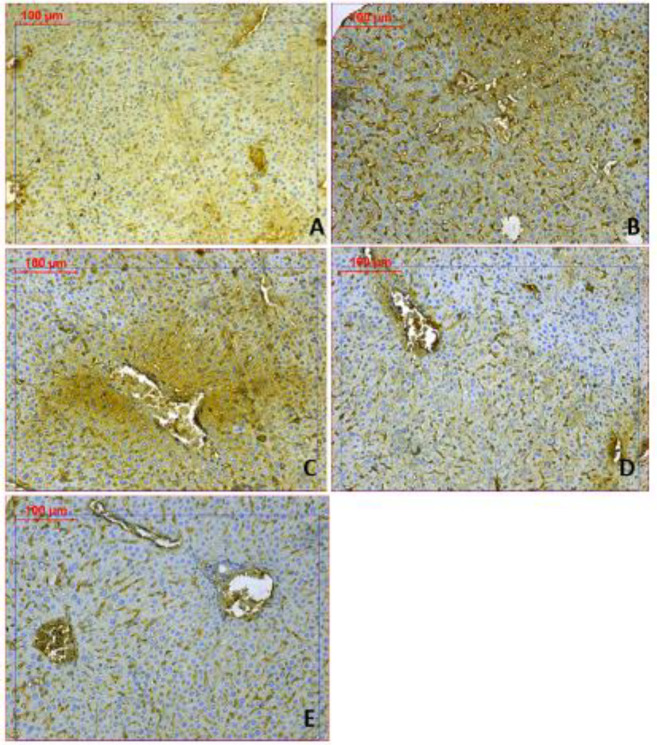
Representative photomicrographs of Bax immunoreactivities in the liver from (A) Vehicle. (B) Rotenone only. (C) Rotenone+amphetamine 0.5 mg/kg. (D) Rotenone+amphetamine 1 mg/kg. (E) Rotenone+amphetamine 2 mg/kg (Bax 200x)

**Figure 12:**
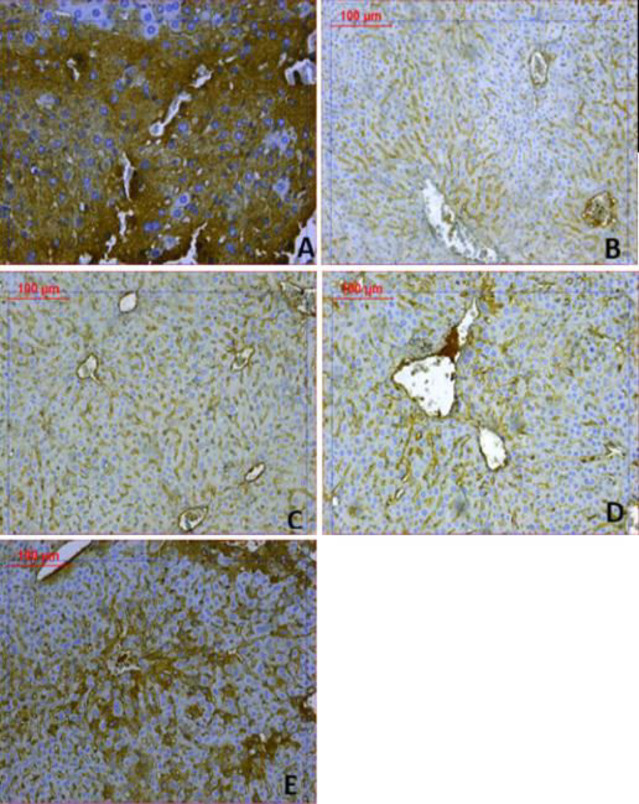
Representative photomicrographs of Bcl2 immunoreactivities in the liver from (A) Vehicle. (B) Rotenone only. (C) Rotenone+amphetamine 0.5 mg/kg. (D) Rotenone+amphetamine 1 mg/kg. (E) Rotenone+amphetamine 2 mg/kg (Bcl2 200x)

Increased expression of Bax protein and minimal expression of Bcl-2 was observed in the rotenone control group, indicating up-regulation of apoptosis (Figures 11&12). Mice treated with amphetamine showed decreased Bax and increased Bcl-2 immunostaining. Quantification of the percentage of cells that showed positive immunostaining for Bax and Bcl-2 is shown in Table 2. Bax/Bcl-2 ratio increased by 92.2% following rotenone injection and decreased by 34.1%, 57.8%, and 89.6% after treatment with different doses of amphetamine.

## Discussion

The results of this study demonstrated that low doses of amphetamine are able to prevent the neurodegenerative changes caused by rotenone in the brain of mice. Our results demonstrate the occurrence of degeneration in most neuronal cells in the substantia nigra after rotenone injection. Neuronal cell loss, degenerative and necrotic changes were also observed in the hippocampus and cerebral cortex. The rotenone-induced pathological changes were prevented by amphetamine, which inhibited tissue oxidative stress and apoptosis. When given at the time of rotenone injection, the drug prevented the loss of neuronal cells in the substantia nigra and the decrease in tyrosine hydroxylase, thereby, suggesting interference with the pathogenetic mechanisms involved in neuronal cell death by the toxicant. One important mechanism is oxidative stress which is considered to mediate nigral cell death in human PD and has been detected in the brain of these patients post-mortem (8, 11). Previous studies demonstrated increased lipid peroxidation and decreased anti-oxidant mechanisms e.g., reduced glutathione, superoxide dismutase, and catalase (39-41) in the rodent brain following the injection of rotenone. Oxidative stress is considered to largely underlie the rotenone-induced dopaminergic cell death, which could be prevented by anti-oxidants e.g., vitamin E (42), vitamin C (43), and the glutathione precursor *N*-acetyl-cysteine (17, 44). Our results showed that amphetamine decreased the lipid peroxidation end product malondialdehyde, indicating decreased free radical attack on membrane lipids. This was accompanied by a significant increase in total anti-oxidant capacity (TAC). The determination of TAC in the plasma or tissues is a widely accepted measure of the anti-oxidant capacity of all anti-oxidants in the sample (45). The observed neuroprotective effect of amphetamine thus involves an anti-oxidant mechanism.

Rotenone induces the expression of both inducible nitric oxide synthase (iNOS) (40) and neuronal NOS (46) in the substantia nigra and striatum and increases nitric oxide in the brains of mice and rats (39, 47, 48). High concentrations of nitric oxide can be neurotoxic. Nitric oxide by reacting with superoxide or molecular oxygen gives rise to peroxynitrite and other reactive oxides of nitrogen e.g., nitrogen dioxide (NO_2_) or dinitrogen trioxide (N_2_O_3_). These species are capable of both oxidation and nitration of tyrosine residues in proteins, and nitrosylation of thiols in proteins or reduced glutathione resulting in neurodegeneration (49, 50). Nitric oxide and reactive nitrogen species have been implicated in the rotenone-induced neurotoxicity and inhibition of NOS by the administration of the neuronal NOS inhibitor 7-nitroimidazole (46) or an iNOS-specific inhibitor (51) has been reported to protect against the rotenone-induced neurotoxicity in rats. Our present results indicate that amphetamine was able to alleviate the increase in brain nitric oxide following rotenone. It, therefore, seems reasonable to suggest that the neuroprotective effect of amphetamine is mediated at least in part through a decrease in intracellular nitric oxide and the consequent oxidative/nitrosative neuronal damage.

Our data provided evidence for an increase in brain levels of the protein transcription factor NF-κB following exposure to rotenone, which is in agreement with previous studies (52). NF-κB is present in the cytosol in an activated form by binding to an inhibitory subunit IκB-α. It is released following inflammatory, toxic, and oxidative signals and translocates to the nucleus, where it induces the expression of several genes encoding inflammatory mediators such as iNOS, cycloxygenase-2, interleukin-1β, interleukin-6, monocytes chemoattractant protein-1, and tumor necrosis factor-α (53). Rotenone induces the expression of iNOS (40), the proinflammatory cytokines tumor necrosis factor-α (47), interleukin-1β (32), and cycloxygenase-2 (44) in the rat brain. These proinflammatory cytokines are known to cause neurodegeneration (54). Rotenone thus might induce the expression of these inflammatory mediators and cytokines by activating NF-κB. Our results showed that the level of NF-κB in the brain of rotenone-treated mice is reduced by amphetamine, thereby, interfering with cytokine-mediated neurodegeneration. Low doses of amphetamine might inhibit NFκB activation directly or as a result of decreased oxidative stress and hence reducing the activation of the NF-κB signaling pathway which is redox-sensitive (55).

We also showed that rotenone decreases paraoxonase-1 (PON-1) activity, which is consistent with other studies (32, 52). The enzyme which possesses an esterase and lactonase activities hydrolyze the active metabolites of a number of organophosphate insecticides. This action is important in view of the evidence linking exposure to these compounds with the increase in the risk for developing PD (56). Studies showed that genetic variation in enzyme activity and decreased expression levels of PON-1 resulted in a decrease in enzyme efficiency to hydrolyze organophosphate insecticides, with consequent increase in toxicity and the risk for developing PD in exposed individuals (57-59). PON-1 possesses both anti-oxidant and anti-inflammatory properties, suggesting that a decrease in PON-1 activity might increase the risk for neurodegenerative diseases (60). In support of this notion are studies showing decreased serum/plasma PON-1 activity in neurological disorders associated with increased oxidative stress and/or neuroinflammation such as autism (61), multiple sclerosis (62), and dementia (63). 

Rotenone induces apoptotic neuronal cell death (17, 64). The apoptotic pathway in the mitochondria is regulated by the Bcl2 family of proteins which includes both the pro-survival or anti-apoptotic Bcl2 proteins and the pro-apoptotic proteins Bcl2 antagonist/killer-1 (Bak), Bcl2-associated X protein (Bax), and BH3 domain-only proteins. Bcl-2 or other anti-apoptotic proteins act to inhibit the activation of Bax and Bak (65). A decrease in Bcl-2 concentration in the striatum and an increase in caspase-3 immunoreactivity in neurons in the striatum, substantia nigra, and cerebral cortex (47, 52) were observed following rotenone injection in rodents. In addition, rotenone caused the activation of Bad, a member of BH3-only (66), and decreased Bcl-2 expression in human dopaminergic cells (67). Our results demonstrate that Bcl-2 protein expression decreased and Bax expression increased in the rotenone only group, whereas amphetamine treatment alleviated these effects, suggesting an antiapoptotic effect for the drug.

The neuroprotective effect of low doses of amphetamine is supported by previous findings in which the drug given at a dose of 2 mg/kg, led to neuroprotection and motor recovery in a stroke model in rats (26). This dose of amphetamine induced the release of DA in caudate and nucleus accumbens in rats (68). The precise mechanism by which amphetamine prevents neuronal injury is not established. There is general agreement that the principal mechanism responsible for the psychostimulant and hyperlocomotor actions of these drugs is DAT-mediated efflux of DA (23, 26). Recent studies, however, have identified novel transporter-independent targets of amphetamines. It has been shown that amphetamines, as well as β-phenylethylamine, some monoamine metabolites, and several drugs affecting monoaminergic transmission, can directly activate specific G protein-coupled trace amine (trace amine 1 [TA1]) receptors with currently unknown functional consequences (69). For example, Sotnikova *et al*. (70) induced severe DA deficiency in mice lacking DAT by administering a tyrosine hydroxylase inhibitor. These mice exhibited severe akinesia, rigidity, and tremor. Surprisingly, high doses of amphetamine and methamphetamine were found to effectively reduce akinesia and rigidity.

Rotenone also causes liver cell injury resulting in vacuolar degeneration, karyolytic and apoptotic hepatocytes, inflammatory cell infiltration (31), and fatty changes (32). Oxidative damage is involved in the rotenone-induced hepatic toxicity. The pesticide was shown to increase lipid peroxidation and nitric oxide and to decrease reduced glutathione in the liver tissue of mice (71). Our present data demonstrate that oxidative stress is involved in rotenone-induced hepto-toxicity and provide evidence for apoptosis as a mechanism underlying hepatocyte injury caused by rotenone. Meanwhile, administration of amphetamine resulted in substantial hepatoprotection by mechanisms involving decreased oxidative stress and apoptosis. 

**Table 1:** Results of immun-morphometric measurements of Bax, Bcl2, and Bax/Bcl2 ratio (apoptotic body index; ABI) for cerebral cortex tissues of different treated groups

**Parameters **	**Bax positive area %**	**Bcl2 positive area%**	**Bax/Bcl2 Ratio (ABI)**
**Groups**			
Vehicle	3.43 ± 0.47	2.98 ± 0.32	1.15
Rotenone	41.66 ± 1.06^*^	6.05 ± 0.39	6.88
Rotenone + amphetamine 0.5 mg/kg	32.9 ± 1.34^*^	4.6 ± 0.81	7.15
Rotenone + amphetamine 1 mg/kg	16.88 ± 1.22^*+^	3.16 ± 0.52^*+^	5.35
Rotenone + amphetamine 2 mg/kg	11.47 ± 0.07^*+^	3.09 ± 1.18^*+^	3.76

Data were expressed as mean±SE. Statistical analysis was carried out by one-way ANOVA. *: *P<*0.05 vs vehicle. +: *P<*0.05 vs rotenone control

**Table 2:** Results of immune-morphometric measurements of Bax, Bcl2, Bax/Bcl2 ratio (apoptotic body index; ABI) for liver tissue of different treated groups

**Parameters **	**Bax positive area %**	**Bcl2 positive area%**	**Bax/Bcl2 Ratio (ABI)**
**Groups**			
Vehicle	6.23 ± 0.09	41.57 ± 1.3	0.1
Rotenone	31.4 ± 0.24^*^	18.06 ± 0.76^*^	1.73
Rotenone + amphetamine 0.5 mg/kg	22.5 ± 0.11^*+^	19.62 ± 1.19^*+^	1.14
Rotenone + amphetamine 1 mg/kg	18.37 ± 0.66^*+^	25.04 ± 1.55^*+^	0.73
Rotenone + amphetamine 2 mg/kg	6.5 ± 1.06^*^	36.02 ± 0.82^*^	0.18

Data were expressed as mean±SE. Statistical analysis was carried out by one-way ANOVA. *: *P<*0.05 vs vehicle. +: *P<*0.05 vs rotenone control

## Conclusion

The present data indicate a protective effect for low dose amphetamine in the rotenone-induced neuro- and hepato-toxicity. These effects are subsequent to a decrease in oxidative stress and apoptosis.
